# Molecular ultrasound imaging of JAM-A depicts early arterial inflammation

**DOI:** 10.18632/aging.101555

**Published:** 2018-09-14

**Authors:** Fabian Kiessling

**Affiliations:** 1 Institute for Experimental Molecular Imaging (ExMI), Helmholtz-Institute for Biomedical Engineering, RWTH Aachen University, Aachen, Germany; 2 Fraunhofer MEVIS, Institute for Medical Image Computing, Aachen, Germany

**Keywords:** molecular imaging, sonography, microbubble, atherosclerosis, cardiovascular

Ultrasound is a workhorse in clinical routine and often the first imaging modality with that a patient gets in contact. Its strengths are real time imaging capability, low costs and lack of radiation exposure. Due to its considerably high user dependence and limited reproducibility, it is not the first choice for whole body screening examinations but favorably suited for monitoring arterial predilection sites for the development of plaques, stenoses, and aneurysms. Here, the assessment of blood flow by Doppler techniques facilitates the differentiation between the vascular wall and the blood. This can be further improved by using gas-filled microbubbles acting as an intravascular contrast agent. The sensitivity of ultrasound to microbubbles is very high and even a single microbubble can be detected. This fact qualifies contrast-enhanced ultrasound imaging for more than just the visualization of the blood. By binding targeting ligands to the surface of microbubbles, they can be turned into molecular imaging probes and used to assess changes in the vascular wall that not yet have an anatomical correlate. There is a long history of molecular ultrasound imaging of the vascular wall including the assessment of thrombosis, inflammation and sclerosis [1], and there is no doubt that the method works reliably. In our previous research on molecular ultrasound imaging, several molecular targets were addressed that may help answering different clinical questions. In this context, the intercellular adhesion molecule-1 (ICAM-1) was presented as a general marker of endothelial inflammation [2]. However, in case of severe vascular damages, where the endothelial layer is partially removed, imaging with ICAM-1 would be difficult since the subendothelial expression is low and thus no signal would be obtained. Here markers are required that are not only expressed on the endothelium but also on the smooth muscle and other stromal cells once they are exposed to the vascular lumen. Here, VCAM-1 [3] and αvβ3 integrin [4] may be ideal targets. As severe damages on the endothelial layer often occur during vascular catheter-based interventions, molecular ultrasound imaging of these markers may facilitate the assessment of vascular injury and healing and thus may help defining the required time period for anticoagulant therapy.

Most of the previously mentioned markers are also upregulated on arteriosclerotic plaques. However, there is still an intense debate, which marker is capable of predicting plaque growth and instability. This information cannot be derived from morphologic imaging and would open important early therapeutic perspectives before stenoses and occlusions occur.

In our recent paper, we evaluated the suitability of the junctional adhesion molecule A (JAM-A) as an early biomarker of vascular inflammation and plaque development using molecular ultrasound imaging and multiphoton microscopy with fluorescent poly(n-butyl cyanoacrylate) (PBCA) microbubbles [5]. JAM-A belongs to an immunoglobulin subclass and is normally located in tight junctions of endothelial cells. Inflammatory stimuli and endothelial activation induce a translocation of JAM-A to the luminal surface, where it mediates leukocyte recruitment and transmigration [6,7]. In line with previous reports indicating that JAM-A is highly responsive to acute changes in blood flow [6,7], we showed in apolipoprotein E–deficient mice that partial ligation of the carotid leads to a very early upregulation of JAM-A expression that can be assessed by the targeted molecular ultrasound probes. Interestingly, a transient increase in JAM-A expression was also observed at the contralateral site, which most probably was induced by the compensatory sudden increase in blood flow and sheer stress. It is noteworthy that additionally to the endothelial translocation of JAM-A, Zhang and co-workers reported an enhanced JAM-A expression by macrophages in vulnerable plaques, which also contributes to the accumulation of targeted microbubbles and may act as a second indicator of vascular inflammation and plaque instability [8].

**Figure 1:**
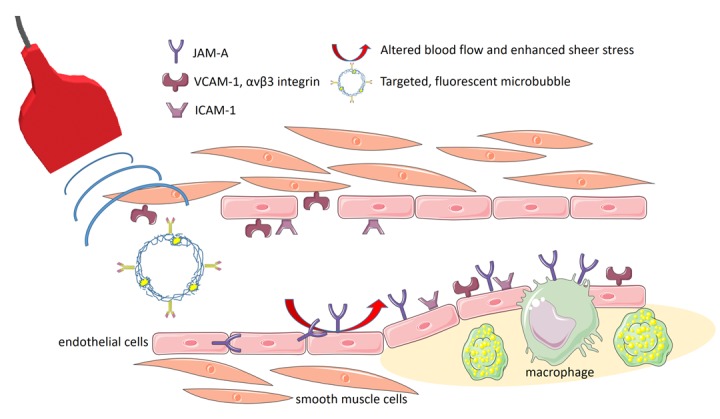
**Scheme illustrating molecular ultrasound imaging with targeted, fluorescent, polymeric ultrasound microbubbles.** While ICAM-1 served as a general target of vascular inflammation, VCAM-1 and αvβ3 faithfully enabled the assessment of major damages of the vascular wall and its regeneration. JAM-A particularly qualified as a very early marker of endothelial activation being translocated to the endothelial surface under altered vascular flow (Clip art was used from the Servier’s “Medical Art” database (http://www.servier.com/Powerpoint-image-bank) and modified).

In summary, we could confirm JAM-A as an interesting biomarker of early vascular inflammation, that may indicate vascular areas exposed to acutely alternated flow and thus links mechanical stress to molecular regulations. Besides for molecular ultrasound imaging, which is more suited to evaluate defined predilection sites, JAM-A targeted probes may also be considered for PET or SPECT imaging, then enabling an efficient whole body staging and the monitoring of therapy, e.g. using statins or inflammatory agents (Figure 1).
